# Quality of life in patients with multiple sclerosis and their caregivers in Colombia: One-year follow-up

**DOI:** 10.7705/biomedica.4759

**Published:** 2020-03-30

**Authors:** Elkin Beltrán, Diana Díaz, Cindy Díaz, Luis Zarco

**Affiliations:** 1 Unidad de Neurología, Hospital Universitario San Ignacio, Pontificia Universidad Javeriana, Bogotá, D.C., Colombia Pontificia Universidad Javeriana Pontificia Universidad Javeriana BogotáD.C Colombia

**Keywords:** Multiple sclerosis, patients, quality of life, caregivers, Colombia, esclerosis múltiple, pacientes, calidad de vida, cuidadores, Colombia

## Abstract

**Introduction:**

Multiple sclerosis is a chronic inflammatory demyelinating disease associated with neurological disability. Clinical features include motor, cerebellar, visual, and sensory function alterations, as well as psychiatric comorbidities, such as depression, anxiety, and irritability. There is little literature available on the quality of life of patients and their caregivers.

**Objective:**

To assess the quality of life of patients with multiple sclerosis and their caregivers in Colombia given that there is no information on the subject in this particular population to establish comprehensive management plans.

**Materials and methods:**

We used the MusiQol and CareQol questionnaires and the Beck Depression Inventory. A prospective analytical observational protocol was designed to include patients aged 18 to 65 years with a diagnosis of relapsing-remitting multiple sclerosis between October, 2014, and October, 2015, at the *Hospital Universitario San Ignacio.* We evaluated quantitative variables and Spearman correlations. The data analysis was carried out with Student t and Mann-Whitney U tests.

**Results:**

A total of 55 patients with relapsing-remitting multiple sclerosis participated in the study. Chronic fatigue was the most common comorbidity in 27%. The MusiQol questionnaire revealed a good basal quality of life, which remained at similar levels in the follow-ups at six and 12 months. Quality of life was good during the study since there were no statistically significant differences between baseline and follow-up MusiQol scores. Good quality of life was also observed in caregivers.

**Conclusions:**

The quality of life of several Colombian patients diagnosed with multiple sclerosis was very good. This positive result was also observed in caregivers as evidenced by the results of the CareQol questionnaire. We also observed and indicated an inversely proportional correlation between the *Expanded Disability Status Scale* and the quality of life indexes.

## Resumen

**Introducción.:**

La esclerosis múltiple es una enfermedad desmielinizante inflamatoria crónica asociada con discapacidad neurológica que afecta la función motora, cerebelosa, visual y sensorial, y se presenta con comorbilidades psiquiátricas como depresión, ansiedad e irritabilidad. Hay escasa literatura disponible sobre la calidad de vida de los pacientes y sus cuidadores.

**Objetivo.:**

Evaluar la calidad de vida de los pacientes con esclerosis múltiple y sus cuidadores en Colombia dado que no hay información sobre el tema en esta población en particular para establecer planes de manejo integral.

**Materiales y métodos.:**

Se utilizaron los cuestionarios MusiQol, CareQol y el inventario de depresión de Beck. Se diseñó un protocolo observacional analítico prospectivo para pacientes de 18 a 65 años con diagnóstico de esclerosis múltiple remitente recurrente entre octubre de 2014 y octubre de 2015 en el Hospital Universitario San Ignacio. Se evaluaron variables cuantitativas y las correlaciones de Spearman. El análisis de datos se hizo mediante la prueba t de Student y la U de Mann-Whitney.

**Resultados.:**

Participaron 55 pacientes diagnosticados con esclerosis múltiple remitente recurrente. En el 27 % de ellos la fatiga crónica fue la comorbilidad más frecuente. El cuestionario MusiQol reveló una buena calidad de vida de base, que se mantuvo en niveles similares en los seguimientos a los seis y los 12 meses. La calidad de vida fue buena durante el estudio y sin diferencias estadísticamente significativas entre las puntuaciones de base del cuestionario MusiQol y las de seguimiento. Se observó una buena calidad de vida en los cuidadores.

**Conclusiones.:**

La calidad de vida de los pacientes colombianos participantes en el estudio con diagnóstico de esclerosis múltiple fue muy buena, así como la de los cuidadores según los resultados del cuestionario CareQol. Se observó una correlación inversamente proporcional entre la *Expanded Disability Status Scale* y los índices de calidad de vida.

Multiple sclerosis is a chronic inflammatory demyelinating disease of the central nervous system associated with neurological disability ^1^. Clinical features frequently include the involvement of the motor, cerebellar, and visual functions, as well as sensory alterations and sphincter dysfunction ^1^^,^^2^. Multiple sclerosis is associated with psychiatric comorbidities; clinical studies list depression, present in up to 50% of patients, besides other frequent psychiatric symptoms including anxiety, irritability, and lability. The prevalence of anxiety in this pathology is 36% ^3^.

Few studies are available regarding the relationship between caregiving family members and the patients, their attitude toward the disease, and the impact on the quality of life of both of them. In Colombia, there are no studies on the current situation of these patients and their caregivers' quality of life. Some studies show a direct link between the severity of the patient's case and the quality of life of the caregiver ^1^^-^^3^. Furthermore, time and money expenses, along with leaves from work, end up affecting the entire family nucleus and, thus, worsening the disease progression. Additionally, the quality of life varies among countries where patients are evaluated depending on sociodemographic factors such as socioeconomic background, level of education, and marital status, which can certainly modify the progression of the disease. A recent multicentric study in Austria, Poland, and Germany showed that each region's unique factors, access to healthcare, beliefs, and customs play a decisive role in the quality of life of multiple sclerosis patients, which varied drastically among the assessed countries ^4^.

In this context, there is a need for studies on the quality of life of Colombian multiple sclerosis patients as there is no information on the matter for this particular population. Data on how these factors affect multiple sclerosis patients are required to establish integral management plans to educate patients and their caregivers based on the needs identified in this study ^4^^-^^6^.

## Materials and methods

Our objective was to evaluate the quality of life of patients with multiple sclerosis and their caregivers in Colombia using the MusiQol and CareQol questionnaires, respectively, to establish comprehensive patient-caregiver management plans.

To measure the quality of life of patients with relapsing-remitting multiple sclerosis in Colombia diagnosed using the criteria described in McDonald, *et al.*^1^, we used the MusiQol and CareQol questionnaires to classify patients and caregivers, respectively. We also sought to collect demographic data including gender, age, ethnicity, level of education, and marital status of both patients and caregivers; measure disability with the Expanded Disability Severity Scale (EDSS); analyze disease-modifying drug therapy adherence and frequency of use, as well as the frequency of depression among patients under study according to the Beck Depression Inventory.

A prospective analytical observational protocol was designed to include patients aged 18 to 65 who met the 2010 McDonald's criteria for relapsing-remitting multiple sclerosis, with EDSS scores for multiple sclerosis between 0 and 6.5 who had been undergoing treatment with a disease-modifying drug for the last six months. Patient exclusion criteria included associated neurological pathologies or systemic diseases, history of alcohol or drug abuse, and multiple sclerosis relapse during the month prior to the evaluation. Between October, 2014, and October, 2015, we collected data from patients with relapsing-remitting multiple sclerosis at the *Hospital Universitario San Ignacio* who met the aforementioned inclusion criteria. A limitation of the study was the small size of the sample, which could render uncertain results.

All participating patients signed an informed consent form approved by the Ethics Committee of the *Pontificia Universidad Javeriana.* Patients accepted into the study filled out a form to collect their demographic information, time of disease course, disease-modifying drugs taken, and drug treatment adherence. We assessed their level of disability at the beginning of the study using the EDSS, their quality of life using the MusiQol questionnaire scale, and their depression using the Beck Depression Inventory. We collected demographic data from caregivers as well and assessed their quality of life using the CareQol questionnaire scale. Patient follow-up was performed six and 12 months after their inclusion in the study using the instruments mentioned above while caregiver follow-up was performed at the same intervals using the CareQol tool.

The MusiQol score quantifies the quality of life of multiple sclerosis patients. The score is directly correlated with the quality of life, i.e., the higher the score, the higher the quality of life of a patient. The MusiQoL questionnaire comprises 31 items in nine dimensions. Each dimension is named according to its constitutive items as follows: activities of daily living (ADL, eight items), psychological well-being (PWB, four items), symptoms (SPT, three items), relationships with friends (RFr, four items), relationships with family (RFa, three items), relationship with the healthcare system (RHCS, three items), sentimental and sexual life (SSL, two items), coping (COP, two items), and rejection (REJ, two items).

Each item was answered using a six-point Likert scale where 1 stood for 'never/not at all', 2 for 'rarely/a little', 3 for 'sometimes/somewhat', 4 for 'often/a lot', 5 for 'always/very much', and 6 for 'not applicable'. The negatively worded item scores were reversed so that higher scores indicated a higher level of health-related quality of life (HRQoL). The patient's score in every one of the dimensions was obtained by computing the mean of the item scores in each dimension. If fewer than half of the items were missing, the mean of the non-missing items was substituted for the missing ones. All dimension scores were linearly transformed to a 0-100 scale. The global index score was computed as the mean of the dimension scores.

The MusiQol questionnaire allows for the evaluation of the quality of life providing a global assessment score. However, it can also be used to measure a variety of specific dimensions. The evaluated domains are activities of daily living, psychological well-being, symptoms, relationships with friends, relationships with family, relationship with the healthcare system, sentimental and sexual life, coping, and rejection.

The CareQol questionnaire allows for the measurement and classification of the quality of life of multiple sclerosis caregivers as excellent, good, fair, and poor; the score is inversely proportional to the caregiver's quality of life, meaning that lower scores reflect better quality of life.

We evaluated continuous quantitative variables using mean and median and handled categorical variables as relative and absolute frequencies. We carried out Spearman correlations between age and questionnaire outcomes and compared means and evaluated data distribution for continuous variables including gender, marital status, occupation, and comorbidities using the Student's t-test for those behaving normally and the Mann-Whitney U-test for those with abnormal distributions. The Kolmogorov-Smirnov test was used to test normality.

## Results

We recruited 55 patients diagnosed with relapsing-remitting multiple sclerosis following the 2010 McDonald's diagnostic criteria ^1^. The average age was 36.2 years, evidencing a 2.1:1 female-to-male ratio. In total, 55% of subjects were mestizos (mixed race) while only 5% were Afro-Latin Americans. Marital status data showed most subjects had stable relationships: 47% were married or living together, while only 13% were divorced. The most frequent level of education attained by subjects was high school, followed by undergraduate education, with 37% and 31%, respectively. Chronic fatigue was the most frequently encountered comorbidity found in 27% of subjects (table 1).

**Table 1:** Symptoms associated with relapsing-remitting multiple sclerosis according to the 2010 McDonald's criteria (N=55)

Comorbidities	n	%
None	19	35
Chronic fatigue	15	27
Anxiety	9	16
Depression	4	7
Insomnia	2	4
All of the above	6	11

As for pathology-related variables, we observed that the average disease duration when entering the study was 4.8 years. Relapses per year averaged 1.1, with a median of 1. The average EDSS score was <3 (better life quality) (table 2).

**Table 2:** Descriptive statistics for disease-related variables (N=55)

Variable	Average	Median	Min	Max
Age (years)	36.2	34	18	60
EDSS	2	1	0	6.5
Time since multiple sclerosis diagnosis (years)	4.8	4	0	13
Relapses per year	1.1	1	0	4

EDSS: Expanded Disability Status Scale score

The most frequent drug therapy among subjects was high-dose interferon beta-1a (42%). Adherence to treatment was 100% (this parameter was established by asking the patients the treatment time and adherence to it). The average treatment duration at the beginning of the study was 44 months.

A discrete progression of disability was observed at the 6-month and 12-month follow-up visits, but it was only one point in the mean (2.25 at six months and twelve months).

The use of these instruments revealed a good baseline quality of life among our patients with a mean of 84.1, which remained at similar levels in the 6-month and 12-month follow-ups (means of 84 and 87.7, respectively). The quality of life was good during the study, as there were no statistically significant differences between baseline and follow-up MusiQol scores (p=0.59).

The baseline quality of life of caregivers was good, with a mean of 32.5, and it remained stable after 6- and 12-month follow-ups with means of 32.6 and 30, respectively (table 3). No statistically significant variation in the quality of life of caregivers was observed along the length of the study compared with baseline CareQol scores (p=0.07). Thus, the quality of life remained stable for multiple sclerosis caregivers as well.

**Table 3:** Baseline and 6- and 12-month CareQol scores for the quality of life of caregivers (N=55)

CareQoL scores	Baseline	Six months	12 months	p value
N	55	55	55	
Mean	32.5	32.6	30	
Min	20	27	20	
Max	53.4	74	54.6	0.07

Values and interpretation: excellent: 20-29; good: 30-39; fair: 40-50, and poor: >50

The CareQol questionnaire also allows for the evaluation of specific quality of life aspects of multiple sclerosis patients' caregivers such as their perception of global health and physical burden, social impact, emotional impact, need for help, and emotional reactions (table 4). We observed a good quality of life in our patients' caregivers in almost every one of these dimensions, as shown by the mean values and baseline averages at the 6- and 12-month follow-ups. Only the emotional impact dimension of the caregivers' quality of life had high scores both at the baseline and during follow-up.

**Table 4:** Baseline and 6- and 12-month CareQol dimension scores for the quality of life of caregivers (closest relative to the patient) (N=55)

CareQoL dimensions	Baseline score	Six months	12 months
	N=55 Mean (min-max)	N=55 Mean (min-max)	N=55 Mean (min-max)
Physical burden/Global health	20 (20-52)	20 (20-60)	20 (20-52.5)
Social impact	20 (20-68)	21 (20-80)	20 (20-55)
Emotional impact	50 (20-86)	48 (20-90)	46.6 (20-86)
Need for help	26 (20-60)	20 (23-73)	20 (20-73.3)
Emotional reactions	20 (20-90)	20 (20-70)	30 (20-60)

Using the Beck inventory we determined the frequency of depression, which had a baseline score of 14% that increased significantly to 22% (p=0.047) at the 6-month follow-up and decreased to 9% after 12 months.

We observed a direct correlation between depression EDSS scores at the beginning of the study in multiple sclerosis patients, as well as an inverse correlation with the patient's age after one year (table 5). We also observed an inverse correlation between caregiver quality of life scores and patient disability at the beginning of the study.

**Table 5:** Spearman's correlation between the patient and caregiver's quality of life, the depression scale and continuous variables of interest

	p value - Age	Correlation coefficient	p value - EDSS	Correlation coefficient
MusiQoL				
Initial	0.518	0.089	0.181	-0.183
Six months	0.159	-0.2.11	0.131	-0.226
One year	0.920	0.019	0.568	-0.109
Beck				
Initial	0.110	-0.218	0.048	0.267
Six months	0.862	0.027	0.156	0.215
One year	0.022	-0.416	0.884	-0.028
CareQoL				
Initial	0.310	0.139	0.001	0.446
Six months	0.562	-0.088	0.335	-0.0145
One year	0.862	-0.029	0.410	0.136

## Discussion

Psychological stress in multiple sclerosis patients is significantly greater than in healthy control subjects and it is an independent predictor of lower quality of life and work productivity, as well as greater costs for the community they interact with. Multiple sclerosis is the most frequently diagnosed demyelinating disease affecting mostly the young productive population. It causes a progressive neurological decline that leads to early disability and unfitness for work ^9^. Few studies have assessed the magnitude of such effects. Kobelt, *et al.* studied 13,186 multiple sclerosis patients in nine European countries reporting that between 33% and 45% of them had accessed an early retirement pension ^10^.

To this date, there is no data available on unemployment rates among multiple sclerosis patients that are fit for work, mostly due to studies focusing on establishing the proportion of economically inactive patients instead. A study ^11^ carried out in Australia established that 50% of males and 75% of female subjects were economically unproductive. Studies show that approximately 50% of patients will require walking aids, psychological treatment, and rehabilitation resulting in a loss of productivity with a great economic impact in terms of health costs and work leaves ^12^.

The chronic and potentially disabling nature of multiple sclerosis constitutes a threat to the marital life of patients. The probability of remaining together on the long-term for an multiple sclerosis patient and his or her partner is 33%, compared to 53% of control subjects, according to a Danish study ^13^; greater divorce rates were observed in men, childless couples, and patients with an onset before 36 years old. Multiple sclerosis significantly affects the partner of the patient when compared to the general population ^14^.

Individuals suffering from this disease progressively develop a compromise of functional capacity due to fatigue. Also, 55% of multiple sclerosis-diagnosed patients describe fatigue as one of the most severe symptoms experienced in the disease. This clinical feature increases the risk of developing depression and affects day-to-day activities, as well as social behavior and quality of life ^15^.

In short, the quality of life of multiple sclerosis patients is affected by many reasons when compared to the general population. Most published studies report a correlation between advanced disability and quality of life. The latter is not the sole important predictor of the affectation suffered by patients of this pathology ^13^. Besides the broad spectrum of symptoms, signs, and functional limitations, multiple sclerosis patients and their families adjust and adapt to major lifestyle changes and many types of restriction. As the disease progresses, the patient loses autonomy and begins to require the presence of caregivers even during everyday activities.

Recently, the role of caregivers in the management of long-term multiple sclerosis patients, as well as the insult and suffering they are subject to has been gaining importance including the analysis of the impact caregiver loss has on the patient's quality of life. Few studies have looked into the relationship between multiple sclerosis patient characteristics and caregiver attributes, which eventually determine the impact on the caregiver's quality of life. The above is essential for early identification of vulnerable caregivers at high risk of compromising their quality of life, a step prior to developing strategies to perform early interventions aimed to improve multiple sclerosis prognosis and avoid caregiver loss ^16^.

In this study we used an assessment tool validated in over 14 languages including Spanish ^7^; its use enabled us to determine a baseline score and the assessment of responses to interventions, as well as the quality of life aspects therapeutic interventions have the greatest impact on.

International research reported a 2.1/1 female-to-male ratio. The low percentage of Afro-Latin American multiple sclerosis patients, only 5%, may be a consequence of the low frequency of multiple sclerosis in that population and/or lower access to healthcare services. The most frequent comorbidity was chronic fatigue, observed in 27% of patients. The time of disease progression for our patients at the moment of inclusion in the study was 4.8 years on average, while 44 months was the average of the duration of treatment with a disease-modifying drug with a 100% adherence. This explains the high functionality associated with low disability at the beginning of the study that we evidenced in a mean score of 1 in the EDSS, which experienced a discrete progression to a mean score of 2 by the end of the follow-up year.

Notwithstanding the methodological design and the low prevalence of multiple sclerosis in Colombia, a total of 3,462 people diagnosed with multiple sclerosis were treated during the 2009-2013 period. The national prevalence for the period was 7.52/100,000 with the highest figures in Bogotá (16.25), where 1,213 patients were attended, followed by the departments of Quindío (13.03) and Risaralda (11.18). Our patient sample entered the study with an EDSS mean score of 1 indicating high functionality and limited disability. This may be due to a relatively short time of disease progression, averaging 4.8 years, but high levels of adherence to treatment with disease-modifying drugs may also play a role in this phenomenon.

The quality of life as measured through the MusiQol questionnaire was very good, with a mean score of 84.1, and statistically insignificant variations at the 6- and 12-month follow-ups (p=0.59). These results can be explained by the functional status and low EDSS score of patients, an early start of disease-modifying drugs, and perfect reported adherence to such treatment, all within a relatively short disease duration of 4.8 years on average at the beginning of the study. Although it was not the objective of this study, other factors such as greater access to information in relation to the disease through the internet could have influenced the results.

Our patient sample had a high quality of life, which remained stable throughout the time of study as evidenced by baseline mean scores and 6-and 12-month follow-up observations.

We measured the quality of life of caregivers employing the CareQol questionnaire reporting good quality of life with a 32.5 mean score; we observed no statistically significant variations (p=0.07) during follow-ups after six and 12 months, which is consistent with the good quality of life and low disability of patients. We also found an inverse correlation between the lower quality of life scores in caregivers with a greater degree of disability in MS patients.

Depression was infrequent among our patients at the beginning of the study, with only 14% presenting this condition; despite increasing to 22% after six months (p=0.047), it remained low in comparison with data from other studies reporting depression in up to 50% of subjects. Our results are most likely due to the fact that our patients were in great functional status as a direct relationship has been described between scores in the Beck Depression Inventory and the EDSS.

This one-year follow-up study revealed that the quality of life of a group of Colombian multiple sclerosis patients meeting the 2010 McDonald's diagnostic criteria was very good as measured with the MusiQol scale. Such a positive outcome was also observed in our patients' caregivers as evidenced by the CareQol scores. We also observed and reported a correlation between greater patient disability scores in the EDSS scale with lower quality of life indexes in the CareQol scale. These findings are consistent with the average time of disease progression of 4.8 years, early treatment with disease-modifying drugs, proper adherence to therapy, and low disability.
